# Editorial: Stem Cells in Tissue Homeostasis and Disease

**DOI:** 10.3389/fcell.2022.876060

**Published:** 2022-03-09

**Authors:** Wencheng Zhang, Yan-Ru Lou, Jianjun Zhou, Zhiying He

**Affiliations:** ^1^ Institute for Regenerative Medicine, Shanghai East Hospital, School of Life Sciences and Technology, Tongji University, Shanghai, China; ^2^ Shanghai Engineering Research Center of Stem Cells Translational Medicine, Shanghai, China; ^3^ Shanghai Institute of Stem Cell Research and Clinical Translation, Shanghai, China; ^4^ Department of Clinical Pharmacy and Drug Administration, School of Pharmacy, Fudan University, Shanghai, China; ^5^ Cancer Stem Cell Institute, Research Center for Translational Medicine, Shanghai East Hospital, Tongji University, Shanghai, China

**Keywords:** stem cell, tissue homeostasis, lineage, cancer stem cell, stem cell-based therapy, dynamic cell tracking, animal model

The regular cellular turning over of organs and tissues throughout life span reveals the homeostasis of an individual in multiple ways, which is commonly disrupted in disease states. Uncovering the potential mechanisms of maintaining and regulating normal status is the key to the development of strategic treatments of disorders and diseases. With the progress of regeneration theory and technology in recent years, the role of tissue-specific stem cells in tissue homeostasis has been increasingly recognized. The potential of stem cell-based cell therapies in restoring organ function and achieving tissue regeneration is fantastic. This Research Topic “Stem cells in Tissue Homeostasis and Disease” published in *Frontiers in Cell and Developmental Biology* explores recent advances in the emerging field of stem cells and regenerative medicine, and tissue engineering with a focus on revealing the mechanisms of tissue homeostasis, which enables us to better understand the causes of diseases and to develop efficient therapeutic strategies.

In order to identify proper treatments of diseases, it is essential to understand the cellular basis of healthy organs. **Stem cells lineage contribution** has been extensively studied during normal embryology development. Hematopoietic stem cells (HSCs) are the best studied stem cells. HSCs maintain the hematopoietic homeostasis via self-renewal and a well-established lineage distribution pattern with blood cell differentiation potentials. This stem cell lineage distribution is not unique to the blood system. Biliary tree stem cells located in peribiliary glands had been proved to participate in the functional regeneration of liver and pancreas. Based on this, Overi et al. had identified a cell compartment with stem/progenitor cell features within pancreatic duct glands (PDGs). These stem/progenitor cells were shown to participate in the islet injury repair in type 2 diabetic mellitus (T2DM) patients and diabetic animal models, indicating that the activation of these somatic stem cells can be a potential strategy for promoting organ regeneration. The same scenario occurs for intestinal stem cells (ISCs). ISCs located in the villi of intestinal crypts and commonly known as Lgr5^+^ subpopulations, are also critical for natural turning over of intestinal mucosa. Song et al. discovered an active fraction of the rhizomes of Trillium tschonoskii Maxim (TT), which can promote irradiated intestinal organoid growth and increase Lgr5^+^ intestinal stem cell numbers, to develop a potential oral drug for improving the regeneration and repair of intestinal epithelia that have intestinal radiation damage.

Besides activating retaining somatic stem cells, other researchers are focusing on targeting the **cancer stem cells (CSCs)**. Chen et al. have summarized the recent studies on the regulation and dysfunction of autophagy-related genes of stem cells which controlled cellular homeostasis of HSCs or leukemia stem cells (LSCs) under different conditions. For the same types of adult stem cells but on a different aspect, Wang et al. reviewed the latest advances to better understand the biological activity of N^6^-methyladenosine (m^6^A), a common modification of mammalian mRNAs, in preserving the function of HSCs and LSCs. Thus, offering the field a promising therapeutic strategy for targeting M^6^A modifiers in myeloid leukemia. Guo et al. discovered miR-221/222 cluster as a novel regulator of CD133+ CSCs in non-small cell lung cancer (NSCLC). Their study not only revealed the Reck-Notch1 signaling as the key mediate of miR-221/222, but also provided a potential therapeutic target for the treatment of NSCLC.

**Stem cell-based therapies** are the most promising strategies for diseases that cannot be treated with traditional surgery or drugs. Various candidate cell types have been explored in recent decades. Use of human pluripotent stem cells (hPSCs), referring to human embryonic stem cells (hESCs) and human induced pluripotent stem cells (iPSCs), in cell therapy still faces their challenges, such as differentiation efficiency, organoid technology, maturity of derived cells, cell expansion, Bogacheva et al. had carefully compared the spheroid size and 3D cell culture systems on spheroid morphology and the effectiveness of definitive endoderm (DE) differentiation, which highlighted the importance of choosing proper biomaterials to achieve successful human iPSC differentiation. Chang et al. have reviewed the most recent advanced development of **hPSCs-derived liver organoids (PSC-LOs)**. Emerging with new bioengineering techniques and tools, PSC-LOs show great potentials in disease modeling, drug development, and liver regeneration.

However, a number of obstacles, such as the lack of an xeno-free expansion system, low yield of *in vitro* differentiation, low maturity, lack of a non-invasive cell tracking method, have slowed the progress of PSC-LOs in clinical applications and drug development. Fortunately, two papers in this Research Topic have made great contribution to overcoming some of these challenges. **Expansion and functional maturation of stem cells** is essential for quality and quantity of cells to meet the need of translational research and clinical applications. Liu et al. reviewed the progress made in the field of *in vitro* generation of platelets from various stem cells, with some achieving large scale production. While cell fate determination post-transplanting or post-grafting has shown its importance for the translation application of stem cells as a therapeutic treatment to various diseases. Advanced technologies in stem cell fate tracking and *in vivo* imaging have been developed over the past decade. However, **dynamic cell tracking** methodology for tracing transplanted cells *in vivo* is still at its early phase. In Lu et al. study, a three-dimensional imaging technique was established to conduct the overall evaluation of transplanted hepatocytes in host liver.

Sharing major biomarkers with hPSCs, human amniotic epithelial stem cells (hAESCs) are preferrable in many clinical studies. Li et al. generated human retinal pigment epithelium (RPE) from hAESCs that had rescued the visual function of a retinal degeneration rat model. Other than hAESCs, mesenchymal stem cells (MSCs) are a commonly used candidate for stem cell-based therapies currently, given their advantages on proliferation, immunomodulation, and tissue maintenance, Yang et al. have reviewed most recent studies in MSC differentiation into specialized cell types, revealing long non-coding RNAs (lncRNAs) as master regulators during the maintenance of homeostasis and multi-differentiation functions through epigenetic, transcriptional, and post-translational mechanisms. Li et al. studied the effect of melatonin on promoting the viability of adipose-derived mesenchymal stem cells (ADMSCs) during the treatment of diabetic mellitus. The melatonin-treated ADMSCs not only performed better in controlling hyperglycemia, insulin resistance, and liver glycogen metabolism in T2DM patients, but also proved to be safe and valuable for pet clinic.

In studies of stem cell-based organ regeneration, **animal models** are critical to study diseases and to develop potential treatments. Chen et al. had established a typical hyperoxia-based Bronchopulmonary dysplasia (BPD) mouse model to mimic hallmarks of BPD, which enabled them to reveal that both cord blood-derived mononuclear cells (CB-MNCs) and umbilical cord-derived mesenchymal stem cells (UC-MSCs) are efficient in alleviating BPD (Chen et al.). In the study of stem cells in the pancreatic ductal glands, Overi et al. evaluated the pancreatic islet injury and regeneration based on the classical streptozotocin (STZ)-induced diabetic mouse model. In the 3D imaging study, Lu et al. conducted the study based on a fumarylacetoacetate hydrolase (Fah) deficiency liver injury model, which enabled them to verify the percentage of cell repopulation of donor cells in host at different time points by using pre-established histology and serology assays.

By focusing on identifying potential candidate stem cells, establishing expansion and maturation systems, and developing grafting technique and post-transplantation cell tracking techniques, the collection of studies in this Research Topic is aiming to promoting the translation of techniques and basic research on stem cells to clinical products.

